# Survival and disease characteristics of de novo versus recurrent metastatic breast cancer in a cohort of young patients

**DOI:** 10.1038/s41416-020-0784-z

**Published:** 2020-03-31

**Authors:** Hayley S. McKenzie, Tom Maishman, Peter Simmonds, Lorraine Durcan, POSH Steering Group, Douglas G. Altman, Louise Jones, Gareth Evans, Alastair M. Thompson, Paul Pharaoh, Andrew Hanby, Sunil Lakhani, Ros Eeles, Fiona J. Gilbert, Hisham Hamed, Shirley Hodgson, Peter Simmonds, Diana Eccles, Diana Eccles, Ellen Copson

**Affiliations:** 1 University Hospital Southampton NHS Foundation Trust, Southampton General Hospital, Southampton, SO16 6YD UK; 2 Southampton Clinical Trials Unit, Southampton General Hospital, Southampton, SO16 6YD UK; 3 Statistics in Medicine, Nuffield Department of Orthopaedics, Rheumatology & Musculoskeletal Sciences, University of Oxford, Oxford, UK; 4 Tumour Biology Department, Institute of Cancer, Barts and The London School of Medicine & Dentistry, London, UK; 5 Genomic Medicine, Division of Evolution and Genomic Sciences, University of Manchester MAHSC, St Mary’s Hospital, Manchester, UK; 6 University of Texas MD Anderson Cancer Center, Houston, TX USA; 7 Public Health and Primary Care, University of Cambridge, Cambridge, UK; 8 Department of Pathology, University of Leeds, Faculty of Medicine, Leeds, UK; 9 Discipline of Molecular & Cellular Pathology, Faculty of Medicine, University of Queensland, The Royal Brisbane & Women’s Hospital, Brisbane, QLD Australia; 10 Institute of Cancer Research, London, UK; 11 Department of Radiology, University of Cambridge, Cambridge Biomedical Campus, Cambridge, UK; 12 Guy’s & St Thomas’ Hospital, London, UK; 13 St George’s Hospital, University of London, London, UK; 14 University Hospital Southampton NHS Foundation Trust, Southampton, UK; 15 Cancer Sciences Academic Unit, Faculty of Medicine, University of Southampton, Southampton, UK

**Keywords:** Breast cancer, Breast cancer, Outcomes research

## Abstract

**Background:**

It is not clear how the pathology, presentation and outcome for patients who present with de novo metastatic breast cancer (dnMBC) compare with those who later develop distant metastases. DnMBC is uncommon in younger patients. We describe these differences within a cohort of young patients in the United Kingdom.

**Methods:**

Women aged 40 years or younger with a first invasive breast cancer were recruited to the prospective POSH national cohort study. Baseline clinicopathological data were collected, with annual follow-up. Overall survival (OS) and post-distant relapse-free survival (PDRS) were assessed using Kaplan–Meier curves.

**Results:**

In total, 862 patients were diagnosed with metastatic disease. DnMBC prevalence was 2.6% (76/2977). Of those with initially localised disease, 27.1% (786/2901) subsequently developed a distant recurrence. Median follow-up was 11.00 years (95% CI 10.79–11.59). Patients who developed metastatic disease within 12 months had worse OS than dnMBC patients (HR 2.64; 1.84–3.77). For PDRS, dnMBC was better than all groups, including those who relapsed after 5 years. Of dnMBC patients, 1.3% had a g*BRCA1*, and 11.8% a g*BRCA2* mutation.

**Conclusions:**

Young women with dnMBC have better PDRS than those who develop relapsed metastatic breast cancer. A gBRCA2 mutation was overrepresented in dnMBC.

## Background

Breast cancer is the most common neoplasm in women, with over 55,000 new diagnoses per year in the United Kingdom.^1^ The vast majority of patients present with disease localised to the breast and axillary lymph nodes, and are treated with the aim of cure, but for the 6–7% who present with de novo metastatic disease (dnMBC), treatment is usually with palliative intent.^1,2^ Overall, the median survival of those with metastatic breast cancer (MBC) is 2–3 years,^3^ although the range is wide, with some patients with ER + or HER2 + disease living much longer. Most MBC survival analyses are retrospective, with a median age of 53–65 (with <15% of participants being aged under 40).^4–6^

A number of studies to date have shown a longer survival time following diagnosis of metastases for those presenting with dnMBC, compared with those who later develop distant metastases after initial treatment for early breast cancer (recurrent MBC, rMBC).^5,7–9^ In a retrospective multicentre study evaluating 815 consecutive patients with MBC in the Netherlands from 2007 to 2009, this was only true for rMBC patients with a metastasis-free interval (MFI) of <24 months.^6^

The phenotype of breast cancer for those with dnMBC is unclear. Compared with rMBC cases, more favourable pathological features have been reported, such as a lower frequency of triple-negative carcinomas.^5,7^ However, more aggressive features have also been documented, such as larger tumours, and an increased frequency that is Grade 3.^5^ However, with median follow-up length of less than 5 years, interpretation of these studies is limited by the omission of late ER + ve recurrences.

Data regarding clinical presentation have also yielded varying results. A higher prevalence of bone involvement in the dnMBC group at diagnosis has been reported in two studies: one reported an equal prevalence of brain metastases, and the other reported fewer brain metastases compared with those with rMBC.^6,7^ Another study also found a lower prevalence of brain metastases, but a similar prevalence of bone involvement.^5^ Locoregional management in patients with dnMBC is the subject of ongoing debate, as results from retrospective studies have been confounded by selection bias, and the results from randomised trials have been conflicting.^10^

Published studies on dnMBC have been limited by their retrospective nature (with the risk of survival bias), or by small patient numbers and short follow-up periods. None of them has complete germline *BRCA* status, or evaluated a specific age group. The incidence of breast cancer in young women (aged <40) is low, but increasing.^11^ Young women are more likely to have breast cancer with adverse biological features, including higher grade, absence of hormone receptors, lymph node involvement and vascular invasion.^12^ Young age has been consistently shown to be an adverse prognostic factor, with a higher risk of distant recurrence.^11^ In addition, this group is less likely to have co-morbidities; they may tolerate chemotherapy and other treatments better than older patients. Although young women present more frequently with stage III disease, dnMBC is found infrequently (1% of those aged under 40 in one retrospective study).^13^ The POSH study, a prospective observational study of almost 3000 patients aged 40 years or younger with a first diagnosis of invasive breast cancer,^12^ provides a unique opportunity to study the natural history of dnMBC in young women. Patients were recruited between 2000 and 2008 in the United Kingdom. A wealth of clinicopathological data is available for these patients, including body mass index and ethnicity, and genotyping for germline *BRCA* mutation status has been performed on the vast majority (> 94%). This is an important variable to study as *BRCA* mutation status is increasingly being incorporated in decision-making regarding optimal treatment.^11^ We aimed to characterise the clinical features, pattern of disease progression and survival of young breast cancer patients who present with metastatic disease, compared with those who later develop distant metastases, in a large prospective cohort genotyped for germline *BRCA1/2*.

## Methods

Prospective outcomes in sporadic vs. hereditary breast cancer (POSH) are a multicentre prospective observational cohort study of young women diagnosed with breast cancer in the United Kingdom. The detailed study protocol was published in 2007.^14^ The study received approval from the South West Multi-Centre Research Ethics Committee (MREC 00/6/69). Written informed consent was obtained from all participants.

### Patients

In total, 3021 female patients were recruited from 127 UK hospitals. Patients were eligible if they were diagnosed with an invasive breast cancer between January 1, 2000 and January 31, 2008, at an age of 40 years or younger. Patients were excluded if they had a previous invasive malignancy (excluding non-melanomatous skin cancer), Patients were consented within 12 months of initial diagnosis. All patients received treatment according to local protocols. Patients with confirmed distant metastatic disease at diagnosis (stage M1) according to the local site comprised the dnMBC cohort. Patients who initially had localised disease (stage M0), but developed distant metastatic disease within the follow-up period, (according to site reporting) comprised the rMBC cohort. Tissue diagnosis of metastatic disease was not mandated by the study. Patients without metastatic disease at any time were not included in this analysis.

### Data collection

Information regarding personal characteristics, tumour pathology, stage and treatment received was collected from medical records at study entry. Family history was collected by questionnaire. Pathology and imaging data were verified with copies of original reports. Follow-up data, including date and site of disease recurrence, were obtained from medical records at 6, 12 months and thereafter annually until death or loss to follow-up. Follow-up interval was determined according to local standards; no imaging or other investigation was mandated by this study, as it was observational. Patients were flagged in the National Health Service Medical Research Information Service to facilitate automatic notification of the date and cause of death. This study presents analyses conducted on follow-up data received until 26 June 2016.

### Biological testing

Oestrogen receptor (ER), progesterone receptor (PR) and HER2 receptor status of primary tumours were determined from routine diagnostic pathology reports. Hormone receptor concentrations equivalent to an Allred score of 3 or more were categorised as positive. Tissue microarray (TMA) immunohistochemical staining was used to supplement missing information regarding receptor status.

DNA for genotyping was extracted from whole-blood samples collected at recruitment. A multiplex amplicon-based library preparation system, Fluidigm Access Array (Fluidigm UK, Cambridge, UK) was used to sequence a panel of breast cancer susceptibility genes, including *BRCA1/2* and *TP53*. Illumina HiSeq2500 next-generation sequencing platform was utilised (Illumina, Little Chesterford, UK). If patients met current UK guidelines for genetic testing, multiplex ligation probe analysis was used to ensure that mutations consisting of large exonic deletions or duplications were not missed. Pathogenic variants were confirmed by Sanger sequencing. Those with variants of unknown significance were classified as *BRCA-*negative.

### Statistical methods

Statistical analyses were performed according to a pre-specified statistical analysis plan (Supplementary Information) as per STROBE guidelines.

The primary objective was the comparison of overall survival (OS) of patients with dnMBC with that of patients with rMBC with a MFI of less than 12 months (early12). OS was defined as the time from the date of diagnosis to death from any cause. MFI was defined as the time from the date of diagnosis to the date of the first distant relapse.

The secondary objectives included the comparison of OS and post-distant relapse survival (PDRS) of patients with dnMBC with that of patients with rMBC with a MFI of less than 24 months (early24). PDRS was defined as time from the date of diagnosis of the first distant metastases (date of diagnosis of primary tumour for patients with dnMBC) to death from any cause. Other secondary objectives included the comparison of PDRS of patients with dnMBC vs. early-12 patients, and the description of clinicopathological features in patients with dnMBC and those with rMBC in four cohorts (recurrent disease within 12 months, within 24 months, between 24 and 60 months and after 60 months). Patient and tumour characteristics included ethnicity, body mass index (BMI), germline *BRCA* status, first site of metastasis and primary tumour grading/receptor status. Time-to-event outcomes were described using Kaplan–Meier curves, and analysed using Cox regression models; stratified Cox models or flexible parametric survival models were used in cases where hazards were time-varying. All multivariable analyses were adjusted for age at diagnosis, BMI, grade, tumour size, pathological N stage, ethnicity and ER and HER2 tumour status. Further objectives included the comparison of OS of dnMBC patients who had surgery (breast-conserving surgery, nodal surgery only or mastectomy) vs. those who had no surgery and assessment of correlation between MFI and PDRS in rMBC patients using the survcorr command in R. Statistical analyses were carried out using Stata v15.1 and RStudio v1.1.456.

The study size and power calculations are discussed in the study protocol.^12^

## Results

A total of 3021 eligible women were recruited to the POSH study. For this study, 44 women were excluded (42 were aged 41–50 years and 2 had missing primary tumour data). Of the 2977 women included, 862 (29.0%) were diagnosed with metastatic disease and comprise the analysis population. There were 76 women (2.6%) who presented with dnMBC. As of June 2016, the distant recurrence rate amongst the 2901 women with localised disease at presentation is 27.1% (*n* = 786). Median follow-up of the analysis population was 11.00 years (95% CI 10.79–11.59, *n* = 862).

Of patients with rMBC, 70 (8.9%) developed metastases within 12 months of diagnosis (early 12). There were 268 women (34.1%), who developed metastatic disease within 24 months of their first diagnosis (early 24), 360 (45.8%) within 24–60 months (early 24–60) and 158 (20.1%) after 60 months (late).

### Baseline clinicopathological data

For the 862 women diagnosed with metastatic disease, clinicopathological data can be seen in Table 1. The proportion of patients that were very young (aged 30 or less) decreased with time to relapse amongst rMBC patients (18.6% for early 12, 6.3% for late relapse). The largest proportion of *BRCA1* mutation carriers was found in the early-24 group (8.6%; 23/268). The largest proportion of *BRCA2* mutation carriers was found in the dnMBC group (11.8%; 9/76); there was only one *BRCA1* mutation in this group (1.3%; 1/76).

**Table 1:** Demographic table by M-stage categories.

	M1 (dnMBC) (*n* = 76)	M0 < 12 months (*n* = 70)	M0 < 24 months (*n* = 268)	M0 24–60 months (*n* = 360)	M0 60+ months (*n* = 158)	Total (*n* = 862)
*Age at diagnosis (years)*						
18–25	4 (5.3%)	0	4 (1.5%)	6 (1.7%)	2 (1.3%)	16 (1.9%)
26–30	6 (7.9%)	13 (18.6%)	37 (13.8%)	37 (10.3%)	8 (5.1%)	88 (10.2%)
31–35	27 (35.5%)	26 (37.1%)	95 (35.4%)	117 (32.5%)	53 (33.5%)	292 (33.9%)
36–40	39 (51.3%)	31 (44.3%)	132 (49.3%)	200 (55.6%)	95 (60.1%)	466 (54.1%)
Total	76 (100%)	70 (100%)	268 (100%)	360 (100%)	158 (100%)	862 (100%)
*Ethnicity*						
Caucasian	67 (88.2%)	60 (85.7%)	236 (89.7%)	328 (91.6%)	136 (86.6%)	767 (89.8%)
Black	6 (7.9%)	7 (10.0%)	17 (6.5%)	18 (5.0%)	13 (8.3%)	54 (6.3%)
Asian	3 (3.9%)	3 (4.3%)	8 (3.0%)	10 (2.8%)	7 (4.5%)	28 (3.3%)
Other	0	0	2 (0.8%)	2 (0.6%)	1 (0.6%)	5 (0.6%)
Total	76 (100%)	70 (100%)	263 (100%)	358 (100%)	157 (100%)	854 (100%)
Missing	0	0	5 (1.9%)	2 (0.6%)	1 (0.6%)	8 (0.9%)
*BMI, categorical*						
Underweight	32 (45.1%)	30 (44.8%)	119 (45.4%)	167 (47.2%)	80 (51.3%)	398 (47.2%)
Overweight	21 (29.6%)	21 (31.3%)	85 (32.4%)	105 (29.7%)	38 (24.4%)	249 (29.5%)
Obese	18 (25.4%)	16 (23.9%)	58 (22.1%)	82 (23.2%)	38 (24.4%)	196 (23.3%)
Total	71 (100%)	67 (100%)	262 (100%)	354 (100%)	156 (100%)	843 (100%)
Missing	5 (6.6%)	3 (4.3%)	6 (2.2%)	6 (1.7%)	2 (1.3%)	19 (2.2%)
*Family history*						
No	48 (68.6%)	45 (67.2%)	180 (68.7%)	238 (68.0%)	106 (68.8%)	572 (68.4%)
Yes	22 (31.4%)	22 (32.8%)	82 (31.3%)	112 (32.0%)	48 (31.2%)	264 (31.6%)
Total	70 (100%)	67 (100%)	262 (100%)	350 (100%)	154 (100%)	836 (100%)
Missing	6 (7.9%)	3 (4.3%)	6 (2.2%)	10 (2.8%)	4 (2.5%)	26 (3.0%)
*Presentation*						
Symptomatic	76 (100.0%)	70 (100.0%)	264 (99.2%)	360 (100.0%)	155 (98.1%)	855 (99.4%)
Screen-detected	0	0	2 (0.8%)	0	1 (0.6%)	3 (0.3%)
Other	0	0	0	0	2 (1.3%)	2 (0.2%)
Total	76 (100%)	70 (100%)	266 (100%)	360 (100%)	158 (100%)	860 (100%)
Missing	0	0	2 (0.7%)	0	0	2 (0.2%)
*BRCA (BRCA1 or 2) status*						
BRCA−	66 (86.8%)	62 (88.6%)	237 (88.4%)	325 (90.3%)	138 (87.3%)	766 (88.9%)
BRCA+	10 (13.2%)	8 (11.4%)	31 (11.6%)	35 (9.7%)	20 (12.7%)	96 (11.1%)
Total	76 (100%)	70 (100%)	268 (100%)	360 (100%)	158 (100%)	862 (100%)
*BRCA1 status*						
BRCA1−	75 (98.7%)	66 (94.3%)	245 (91.4%)	346 (96.1%)	148 (93.7%)	814 (94.4%)
BRCA1+	1 (1.3%)	4 (5.7%)	23 (8.6%)	14 (3.9%)	10 (6.3%)	48 (5.6%)
Total	76 (100%)	70 (100%)	268 (100%)	360 (100%)	158 (100%)	862 (100%)
*BRCA2 status*						
BRCA2−	67 (88.2%)	66 (94.3%)	260 (97.0%)	339 (94.2%)	148 (93.7%)	814 (94.4%)
BRCA2+	9 (11.8%)	4 (5.7%)	8 (3.0%)	21 (5.8%)	10 (6.3%)	48 (5.6%)
Total	76 (100%)	70 (100%)	268 (100%)	360 (100%)	158 (100%)	862 (100%)
*TP53 status*						
TP53–	76 (100.0%)	69 (98.6%)	267 (99.6%)	359 (99.7%)	158 (100.0%)	860 (99.8%)
TP53+	0	1 (1.4%)	1 (0.4%)	1 (0.3%)	0	2 (0.2%)
Total	76 (100%)	70 (100%)	268 (100%)	360 (100%)	158 (100%)	862 (100%)
*Histological grade*						
1	2 (2.9%)	0	1 (0.4%)	10 (2.8%)	6 (3.9%)	19 (2.3%)
2	23 (33.8%)	9 (13.0%)	42 (16.0%)	123 (35.0%)	74 (48.7%)	262 (31.4%)
3	43 (63.2%)	60 (87.0%)	220 (83.7%)	218 (62.1%)	72 (47.4%)	553 (66.3%)
Total	68 (100%)	69 (100%)	263 (100%)	351 (100%)	152 (100%)	834 (100%)
Missing	8 (10.5%)	1 (1.4%)	5 (1.9%)	9 (2.5%)	6 (3.8%)	28 (3.2%)
*Histological type*						
Ductal	65 (86.7%)	68 (97.1%)	239 (89.8%)	308 (86.8%)	136 (87.2%)	748 (87.8%)
Ductal and lobular	4 (5.3%)	2 (2.9%)	7 (2.6%)	12 (3.4%)	4 (2.6%)	27 (3.2%)
Lobular	3 (4.0%)	0	6 (2.3%)	21 (5.9%)	14 (9.0%)	44 (5.2%)
Medullary	0	0	2 (0.8%)	2 (0.6%)	0	4 (0.5%)
Metaplastic	0	0	4 (1.5%)	0	0	4 (0.5%)
Mixed	2 (2.7%)	0	4 (1.5%)	4 (1.1%)	0	10 (1.2%)
Other	1 (1.3%)	0	4 (1.5%)	8 (2.3%)	2 (1.3%)	15 (1.8%)
Total	75 (100%)	70 (100%)	266 (100%)	355 (100%)	156 (100%)	852 (100%)
Missing	1 (1.3%)	0	2 (0.7%)	5 (1.4%)	2 (1.3%)	10 (1.2%)
*Surgical margins (mm)*						
0	5 (12.2%)	6 (10.0%)	27 (11.9%)	33 (12.0%)	15 (11.7%)	80 (11.9%)
>0 to <1	0	3 (5.0%)	7 (3.1%)	3 (1.1%)	3 (2.3%)	13 (1.9%)
>=1 to < =5	19 (46.3%)	36 (60.0%)	115 (50.9%)	155 (56.4%)	66 (51.6%)	355 (53.0%)
>5	17 (41.5%)	15 (25.0%)	77 (34.1%)	84 (30.5%)	44 (34.4%)	222 (33.1%)
Total	41 (100%)	60 (100%)	226 (100%)	275 (100%)	128 (100%)	670 (100%)
Missing	35 (46.1%)	10 (14.3%)	42 (15.7%)	85 (23.6%)	30 (19.0%)	192 (22.3%)
*Lymphovascular invasion*						
Absent	18 (31.0%)	15 (23.1%)	68 (27.4%)	121 (35.6%)	60 (41.1%)	267 (33.7%)
Present	40 (69.0%)	50 (76.9%)	180 (72.6%)	219 (64.4%)	86 (58.9%)	525 (66.3%)
Total	58 (100%)	65 (100%)	248 (100%)	340 (100%)	146 (100%)	792 (100%)
Missing	18 (23.7%)	5 (7.1%)	20 (7.5%)	20 (5.6%)	12 (7.6%)	70 (8.1%)
*Path T stage*						
T0	1 (2.1%)	1 (1.5%)	7 (2.7%)	7 (2.0%)	3 (1.9%)	18 (2.2%)
T1	16 (33.3%)	16 (23.9%)	79 (30.6%)	136 (38.7%)	68 (43.9%)	299 (36.8%)
T2	22 (45.8%)	34 (50.7%)	128 (49.6%)	168 (47.9%)	70 (45.2%)	388 (47.8%)
T3	9 (18.8%)	14 (20.9%)	41 (15.9%)	38 (10.8%)	14 (9.0%)	102 (12.6%)
T4	0	2 (3.0%)	3 (1.2%)	2 (0.6%)	0	5 (0.6%)
Total	48 (100%)	67 (100%)	258 (100%)	351 (100%)	155 (100%)	812 (100%)
Missing	28 (36.8%)	3 (4.3%)	10 (3.7%)	9 (2.5%)	3 (1.9%)	50 (5.8%)
*Max tumour size (invasive) (mm)*						
Median	35	32	30	27	25	28
Range	2–80	3–160	2–160	0–199	.5–102	0–199
IQR^a^	18–49	25–60	20–47	18–41	18–40	19–43
Missing	29 (38.2%)	5 (7.1%)	18 (6.7%)	21 (5.8%)	6 (3.8%)	74 (8.6%)
*No. of positive lymph nodes*						
0	11 (23.4%)	11 (16.2%)	66 (25.5%)	96 (26.8%)	49 (31.4%)	222 (27.1%)
1–3	12 (25.5%)	21 (30.9%)	89 (34.4%)	128 (35.8%)	70 (44.9%)	299 (36.5%)
4–9	11 (23.4%)	19 (27.9%)	52 (20.1%)	86 (24.0%)	30 (19.2%)	179 (21.8%)
10+	13 (27.7%)	17 (25.0%)	52 (20.1%)	48 (13.4%)	7 (4.5%)	120 (14.6%)
Total	47 (100%)	68 (100%)	259 (100%)	358 (100%)	156 (100%)	820 (100%)
Missing	29 (38.2%)	2 (2.9%)	9 (3.4%)	2 (0.6%)	2 (1.3%)	42 (4.9%)
*ER status*						
Negative	23 (30.7%)	41 (58.6%)	146 (54.7%)	99 (27.6%)	25 (15.8%)	293 (34.1%)
Positive	52 (69.3%)	29 (41.4%)	121 (45.3%)	260 (72.4%)	133 (84.2%)	566 (65.9%)
Total	75 (100%)	70 (100%)	267 (100%)	359 (100%)	158 (100%)	859 (100%)
Missing	1 (1.3%)	0	1 (0.4%)	1 (0.3%)	0	3 (0.3%)
*PR status*						
Negative	24 (40.7%)	41 (66.1%)	161 (70.6%)	122 (41.9%)	27 (21.8%)	334 (47.6%)
Positive	35 (59.3%)	21 (33.9%)	67 (29.4%)	169 (58.1%)	97 (78.2%)	368 (52.4%)
Total	59 (100%)	62 (100%)	228 (100%)	291 (100%)	124 (100%)	702 (100%)
Missing	17 (22.4%)	8 (11.4%)	40 (14.9%)	69 (19.2%)	34 (21.5%)	160 (18.6%)
*HER2 status*						
Negative	38 (52.1%)	44 (62.9%)	177 (67.6%)	224 (66.3%)	106 (75.7%)	545 (67.0%)
Positive	35 (47.9%)	26 (37.1%)	85 (32.4%)	114 (33.7%)	34 (24.3%)	268 (33.0%)
Total	73 (100%)	70 (100%)	262 (100%)	338 (100%)	140 (100%)	813 (100%)
Missing	3 (3.9%)	0	6 (2.2%)	22 (6.1%)	18 (11.4%)	49 (5.7%)
*TNBC status*^b^						
Not TNBC	72 (94.7%)	46 (65.7%)	168 (62.7%)	305 (84.7%)	143 (90.5%)	688 (79.8%)
TNBC	4 (5.3%)	24 (34.3%)	100 (37.3%)	55 (15.3%)	15 (9.5%)	174 (20.2%)
Total	76 (100%)	70 (100%)	268 (100%)	360 (100%)	158 (100%)	862 (100%)
Missing	0	0	0	0	0	0
*Focality*						
Localised	30 (61.2%)	40 (63.5%)	161 (66.5%)	202 (61.6%)	89 (64.5%)	482 (63.7%)
Multifocal	19 (38.8%)	23 (36.5%)	81 (33.5%)	126 (38.4%)	49 (35.5%)	275 (36.3%)
Total	49 (100%)	63 (100%)	242 (100%)	328 (100%)	138 (100%)	757 (100%)
Missing	27 (35.5%)	7 (10.0%)	26 (9.7%)	32 (8.9%)	20 (12.7%)	105 (12.2%)
*Surgical type*						
BCS	16 (21.1%)	18 (25.7%)	97 (36.2%)	120 (33.3%)	59 (37.3%)	292 (33.9%)
Mastectomy	33 (43.4%)	50 (71.4%)	166 (61.9%)	238 (66.1%)	98 (62.0%)	535 (62.1%)
Nodal surgery only	1 (1.3%)	0	0	1 (0.3%)	0	2 (0.2%)
None	26 (34.2%)	2 (2.9%)	5 (1.9%)	1 (0.3%)	1 (0.6%)	33 (3.8%)
Total	76 (100%)	70 (100%)	268 (100%)	360 (100%)	158 (100%)	862 (100%)
*Neoadjuvant chemotherapy*^c^						
Yes	0	28 (40.0%)	87 (32.5%)	84 (23.3%)	26 (16.5%)	197 (22.9%)
No	76 (100%)	42 (60%)	181 (67.5%)	276 (76.7%)	132 (83.5%)	665 (77.1%)
Total	76 (100%)	70 (100%)	268 (100%)	360 (100%)	158 (100%)	862 (100%)
*Adjuvant chemotherapy*						
Yes	0	40 (57.1%)	173 (64.6%)	256 (71.1%)	120 (75.9%)	549 (63.7%)
No	76 (100%)	30 (42.9%)	95 (35.4%)	104 (28.9%)	38 (24.1%)	313 (36.3%)
Total	76 (100%)	70 (100%)	268 (100%)	360 (100%)	158 (100%)	862 (100%)
*Palliative chemotherapy*						
Yes	75 (98.7%)	50 (71.4%)	204 (76.1%)	301 (83.6%)	111 (70.3%)	691 (80.2%)
No	1 (1.3%)	20 (28.6%)	64 (23.9%)	59 (16.4%)	47 (29.7%)	171 (19.8%)
Total	76 (100%)	70 (100%)	268 (100%)	360 (100%)	158 (100%)	862 (100%)
*Palliative trastuzumab*						
Yes	12 (15.8%)	6 (8.6%)	19 (7.1%)	36 (10.0%)	10 (6.3%)	77 (8.9%)
No	64 (84.2%)	64 (91.4%)	249 (92.9%)	324 (90.0%)	148 (93.7%)	785 (91.1%)
Total	76 (100%)	70 (100%)	268 (100%)	360 (100%)	158 (100%)	862 (100%)
*Palliative radiotherapy*						
Yes	58 (76.3%)	42 (60.0%)	155 (57.8%)	205 (56.9%)	79 (50.0%)	497 (57.7%)
No	18 (23.7%)	28 (40.0%)	113 (42.2%)	155 (43.1%)	79 (50.0%)	365 (42.3%)
Total	76 (100%)	70 (100%)	268 (100%)	360 (100%)	158 (100%)	862 (100%)
*Hormone treatment*						
Yes	52 (68.4%)	20 (28.6%)	80 (29.9%)	169 (46.9%)	97 (61.4%)	398 (46.2%)
No	24 (31.6%)	50 (71.4%)	188 (70.1%)	191 (53.1%)	61 (38.6%)	464 (53.8%)
Total	76 (100%)	70 (100%)	268 (100%)	360 (100%)	158 (100%)	862 (100%)
*Site of metastases at any time*						
Bone	8 (10.5%)	7 (10.0%)	31 (12.0%)	31 (8.9%)	14 (10.0%)	84 (10.2%)
Bone–Brain	4 (5.3%)	4 (5.7%)	7 (2.7%)	13 (3.7%)	2 (1.4%)	26 (3.2%)
Bone–Visc^d^	22 (28.9%)	12 (17.1%)	84 (32.4%)	128 (36.8%)	65 (46.4%)	299 (36.3%)
Bone–Visc–Brain	20 (26.3%)	15 (21.4%)	41 (15.8%)	68 (19.5%)	19 (13.6%)	148 (18.0%)
Brain	3 (3.9%)	4 (5.7%)	18 (6.9%)	11 (3.2%)	5 (3.6%)	37 (4.5%)
Nodal	4 (5.3%)	3 (4.3%)	8 (3.1%)	15 (4.3%)	10 (7.1%)	37 (4.5%)
Visc	12 (15.8%)	21 (30.0%)	53 (20.5%)	55 (15.8%)	17 (12.1%)	137 (16.6%)
Visc–Brain	3 (3.9%)	4 (5.7%)	17 (6.6%)	27 (7.8%)	8 (5.7%)	55 (6.7%)
Total	76 (100%)	70 (100%)	259 (100%)	348 (100%)	140 (100%)	823 (100%)
Missing	0	0	9 (3.4%)	12 (3.3%)	18 (11.4%)	39 (4.5%)
*Site of first metastases*						
Bone	23 (30.3%)	16 (22.9%)	52 (20.1%)	73 (21.1%)	30 (21.7%)	178 (21.7%)
Bone–Brain	0	2 (2.9%)	4 (1.5%)	6 (1.7%)	0	10 (1.2%)
Bone–Visc	20 (26.3%)	12 (17.1%)	73 (28.2%)	108 (31.2%)	58 (42.0%)	259 (31.6%)
Bone–Visc–Brain	0	5 (7.1%)	13 (5.0%)	22 (6.4%)	2 (1.4%)	37 (4.5%)
Brain	1 (1.3%)	4 (5.7%)	18 (6.9%)	13 (3.8%)	5 (3.6%)	37 (4.5%)
Nodal	12 (15.8%)	4 (5.7%)	17 (6.6%)	23 (6.6%)	15 (10.9%)	67 (8.2%)
Visc	20 (26.3%)	25 (35.7%)	73 (28.2%)	88 (25.4%)	23 (16.7%)	204 (24.9%)
Visc–Brain	0	2 (2.9%)	9 (3.5%)	13 (3.8%)	5 (3.6%)	27 (3.3%)
Total	76 (100%)	70 (100%)	259 (100%)	346 (100%)	138 (100%)	819 (100%)
Missing	0	0	9 (3.4%)	14 (3.9%)	20 (12.7%)	43 (5.0%)
*Brain metastases at any time*						
Yes	30 (39.5%)	27 (38.6%)	83 (32.0%)	119 (34.2%)	34 (24.3%)	266 (32.3%)
No	46 (60.5%)	43 (61.4%)	176 (68.0%)	229 (65.8%)	106 (75.7%)	557 (67.7%)
Total	76 (100%)	70 (100%)	259 (100%)	348 (100%)	140 (100%)	823 (100%)
Missing	0	0	9 (3.4%)	12 (3.3%)	18 (11.4%)	39 (4.5%)
*Bone metastases at any time*						
Yes	54 (71.1%)	38 (54.3%)	163 (62.9%)	240 (69.0%)	100 (71.4%)	557 (67.7%)
No	22 (28.9%)	32 (45.7%)	96 (37.1%)	108 (31.0%)	40 (28.6%)	266 (32.3%)
Total	76 (100%)	70 (100%)	259 (100%)	348 (100%)	140 (100%)	823 (100%)
Missing	0	0	9 (3.4%)	12 (3.3%)	18 (11.4%)	39 (4.5%)
*Visceral metastases at any time*						
Yes	57 (75.0%)	52 (74.3%)	195 (75.3%)	278 (79.9%)	109 (77.9%)	639 (77.6%)
No	19 (25.0%)	18 (25.7%)	64 (24.7%)	70 (20.1%)	31 (22.1%)	184 (22.4%)
Total	76 (100%)	70 (100%)	259 (100%)	348 (100%)	140 (100%)	823 (100%)
Missing	0	0	9 (3.4%)	12 (3.3%)	18 (11.4%)	39 (4.5%)

^a^IQR, interquartile range.

^b^TNBC, triple-negative breast cancer.

^c^Chemotherapy refers to cytotoxic chemotherapy only, not hormone, antibody or other therapy.

^d^Visc, visceral metastatic disease.

On review of tumour characteristics, ER positivity was positively correlated with later time of relapse: 45.3% of early 24, 72.4% of early 24–60 and 84.2% of late-relapse cases (69.3% of dnMBC). HER2 positivity was the highest in the dnMBC cohort with 47.9% vs. 32.4% in the early-24 group, and 24.3% in the late-relapse group. The median maximum tumour size was the largest in the dnMBC group at 35 mm, compared with 25 mm in the late-relapse group. The proportion of cases with Grade 3 disease was inversely proportional with time to relapse amongst the rMBC group: 87.0% of early 12 and 47.4% of the late-relapse group (63.2% of dnMBC).

The early-12 group had a high proportion of adverse tumour characteristics, including Grade 3 disease (87.0%), LVI (76.9%) and node positivity (83.8%). The number of involved lymph nodes was more than 10 in a quarter of cases (25.0%), and over a third of cases were triple negative (34.3%).

### Survival

**Fig. 1:**
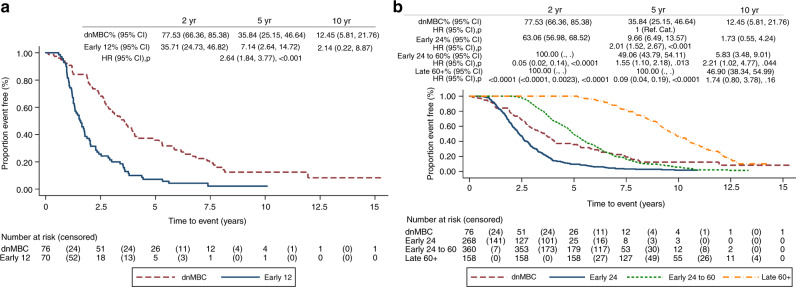
Kaplan-Meier plots of overall survival. **a** OS for dnMBC vs. early 12, reference category: dnMBC. **b** OS for dnMBC vs. early 24, early 24– 60 and late60 + , reference category: dnMBC.

**Fig. 2:**
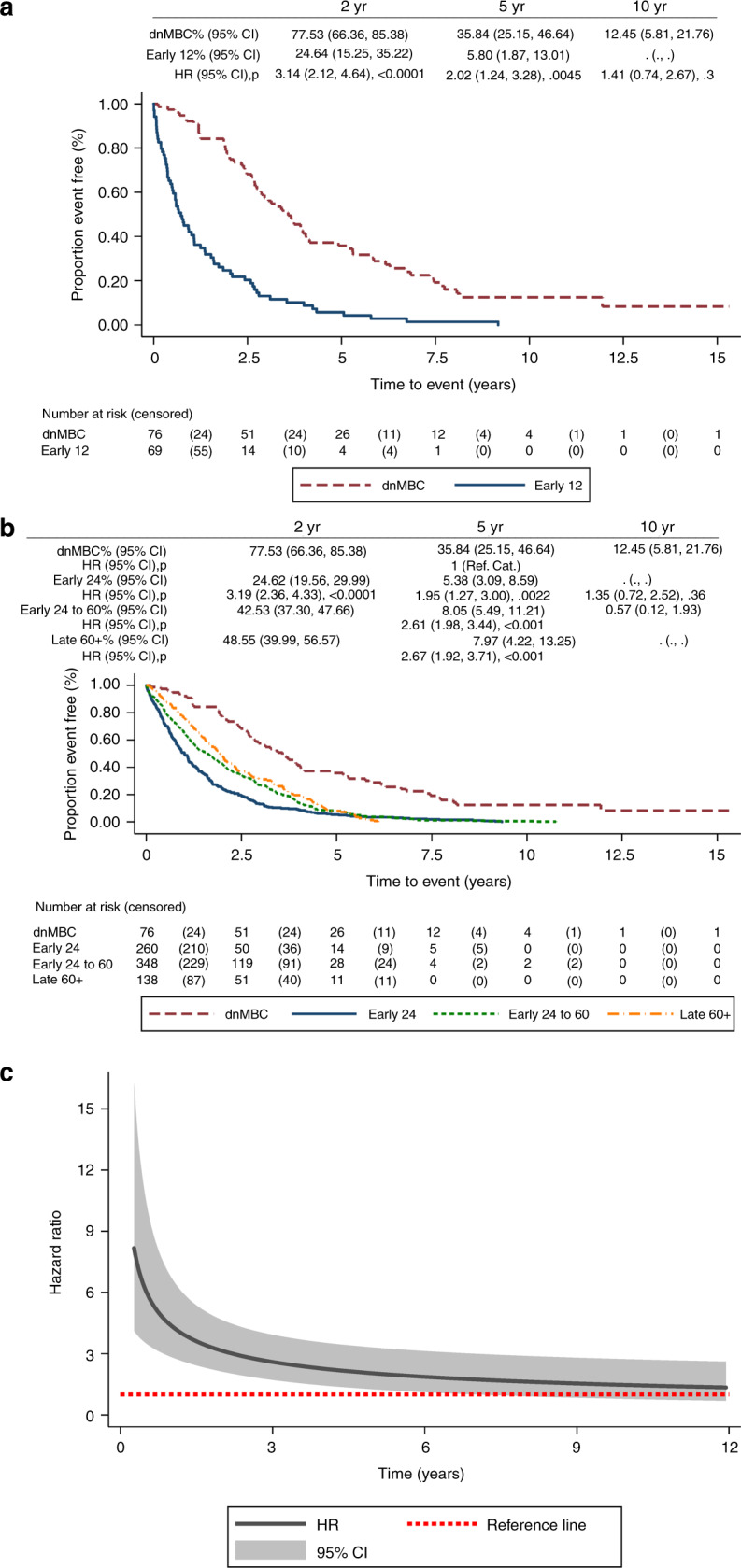
Kaplan-Meier plots of post-distant relapse-free survival. **a** PDRS for dnMBC vs. early 12, reference category: dnMBC. **b** PDRS for dnMBC vs. early 24, early 24–60 and early 60 + , reference category: dnMBC. **c** Time-varying HR for PDRS for dnMBC vs. early12, reference category: dnMBC.

Patients who relapsed within 12 months had a significantly worse OS than the dnMBC group (Fig. 1a), with a HR for death of 2.64 (1.84–3.77; *p* = < 0.001). For those who relapsed after 24 months, the OS varied over time, consistent with the delay from diagnosis to metastatic disease (clearly the HR for death at 2 years was very small). However, results from the time-varying regression model show that by 5 years, the risk of death for those who relapsed between 24 and 60 months was increased, compared with the dnMBC group, with a 5-year HR of 1.55 (1.10–2.18, *p* = 0.013) and 10-year HR 2.21 (1.02–4.77, *p* = 0.044) (Fig. 1b; Supplementary S1A). Similarly, the time-varying regression model shows that, for those who relapsed after 60 months, the risk of death at 5 years was very small, but compared with the dnMBC group, the 10-year HR was 1.74 (0.80–3.78, *p* = 0.160) (Fig. 1b; Supplementary S1B). There was longer PDRS for dnMBC compared with all other groups who developed metastases, including those with late relapse after 60 months (HR 2.67; 1.92–3.71, *p* < 0.001) (Fig. 2a, b). The hazard ratio for PDRS for early 12, compared with dnMBC, decreased over time (Fig. 2c).

We assessed for a correlation between time from initial diagnosis to metastatic relapse (metastasis-free interval, MFI) and PDRS, and found a very slight positive Rho correlation coefficient of 0.045 (95% CI: −0.023 to 0.113), suggesting that there is not a close correlation between these two factors.

A multivariable analysis was performed to assess for factors related to duration of survival in those with dnMBC vs. early12. For OS (Table 2), early-12 patients maintained a significantly worse OS compared with dnMBC after adjustment for other factors (HR 3.76; 2.22–6.38; *p* < 0.001). Positive nodes were found to be associated with significantly shorter survival (HR 2.29; 1.17–4.47; *p* = 0.015), whilst patients with HER2-positive tumours were at reduced risk of death (HR 0.500; 0.311–0.802; *p* = 0.004). Similar results were also found in the multivariable analyses for PDRS.

**Table 2:** MVA^a^ for OS and PDRS for dnMBC vs. early 12.

	OS	PDRS
Covariate	HR (95% CI), *p* value	
*DENOVO*		
dnMBC	1 (Ref. category)	1 (Ref. category)
Early 12	3.76 (2.22–6.38), <0.001	5.12 (2.95–8.87), <0.001
Age at diagnosis (years)	0.97 (0.91–1.03), 0.295	0.97 (0.91–1.03), 0.355
*BMI*		
BMI < 25	1 (Ref. category)	1 (Ref. category)
25 ≤ BMI < 30	1.31 (0.76–2.25), *p* = 0.333	1.27 (0.74–2.20), *p* = 0.387
BMI ≥ 30	1.30 (0.75–2.27), *p* = 0.355	1.21 (0.69–2.12), *p* = 0.498
*Grade*		
1	1 (Ref. category)	1 (Ref. category)
2	7.78 (0.69–87.14), *p* = 0.096	7.14 (0.65–78.91), *p* = 0.109
3	5.47 (0.54–55.22), *p* = 0.150	5.13 (0.51–51.75), *p* = 0.165
Max invasive size (mm)	1.00 (0.99–1.01), *p* = 0.997	1.00 (0.99–1.01), *p* = 0.900
*N stage*		
N0	1 (Ref. category)	1 (Ref. category)
N1	2.29 (1.17–4.47), *p* = 0.015	2.42 (1.23–4.77), *p* = 0.011
*HER2 status*		
Negative	1 (Ref. category)	1 (Ref. category)
Positive	0.50 (0.31–0.80), *p* = 0.004	0.48 (0.30–0.78), *p* = 0.003

^a^MVA Cox model stratified by ER status and ethnicity due to time-varying hazards.

For early-24 patients (Table 3), PDRS was worse compared with dnMBC patients after adjustment for other factors at 2 and 5 years (HR 2.53; 1.50–4.27; *p* < 0.001, and HR 2.42; 1.39–4.22; *p* = 0.0019). Again, positive nodes were found to be a significant risk of earlier distant relapse (HR 1.42; 1.05–1.93; *p* = 0.024), whilst patients with HER2-positive tumours had longer survival (HR 0.66; 0.51–0.86; *p* = 0.002). ER-positive status was protective for disease relapse at 2 years compared with ER-negative (HR 0.50; 0.38–0.67; *p* < 0.001), but not at 5 or 10 years.

**Table 3:** MVA^a^ for PDRS for dnMBC vs. early 24.

Covariate	HR (95% CI), *p* value
*DENOVO (time-varying)*	
dnMBC	1 (Ref. category)
Early 24 (at 2 years)	2.53 (1.50–4.27), 0.00052
Early 24 (at 5 years)	2.42 (1.39–4.22), 0.0019
Early 24 (at 10 years)	3.88 (1.07–14.07), 0.039
Age at diagnosis (years)	1.01 (0.98–1.05), 0.458
*BMI*	
BMI < 25	1 (Ref. category)
25 ≤BMI < 30	1.14 (0.84–1.53), *p* = 0.403
BMI ≥ 30	1.19 (0.86–1.65), *p* = 0.301
*Grade*	
1	1 (Ref. category)
2	1.09 (0.24–4.89), *p* = 0.912
3	1.03 (0.23–4.51), *p* = 0.971
Max invasive size (mm)	1.00 (0.997–1.01), *p* = 0.384
*N stage*	
N0	1 (Ref. category)
N1	1.42 (1.05–1.93), *p* = 0.024
*HER2 status*	
Negative	1 (Ref. category)
Positive	0.66 (0.51–0.86), *p* = 0.002
*Ethnicity*	
White/Caucasian	1 (Ref. category)
Black	0.61 (0.34–1.12), *p* = 0.114
Asian	1.63 (0.74–3.61), *p* = 0.225
Other	0.43 (0.10–1.77), *p* = 0.240
*ER status (time-varying)*	
Negative	1 (Ref. category)
Positive (at 2 years)	0.50 (0.38–0.67), *p* < 0.0001
Positive (at 5 years)	0.79 (0.48–1.29), *p* = 0.350
Positive (at 10 years)	1.30 (0.52–3.25), *p* = 0.590

^a^Stpm2 model (Flexible parametric survival model). Time varying for dnMBC vs. early24 and ER status. However, no stratification of ethnicity incorporated into this model as non-time-varying.

### Sites of metastases

Regarding sites of metastases (at any time during disease course), patients in the dnMBC and early-12 groups were most likely to have widespread (bone, visceral and brain) disease, with 26.3% and 21.4%, respectively, compared with 13.6% in the late-relapse group. Patients with dnMBC had the highest prevalence of brain metastases (39.5%), which decreased with time to relapse (24.3% in the late-relapse group). The proportion with bone metastases correlated with time to relapse amongst those with rMBC: 54.3% of the early 12, up to 71.4% with late relapse (71.1% of the dnMBC group). Visceral metastases were equally prevalent throughout all groups.

When the first site of metastases was evaluated, bone-only or nodal-only disease at presentation was most common in the dnMBC group (30.3% and 15.8%, respectively). Visceral metastases at presentation of metastatic disease were less common in the dnMBC group (52.6%), and most common in the early 24–60 group (66.8%). Bony metastases at presentation were present in 50% of the early-12 group, increasing to 60.4% of the late- relapse group (57% of dnMBC). Brain metastases at presentation decreased with time to relapse amongst the rMBC cohort: 18.6% of early 12 and 8.7% of late relapses (1.3% of dnMBC).

### Treatment

**Fig. 3:**
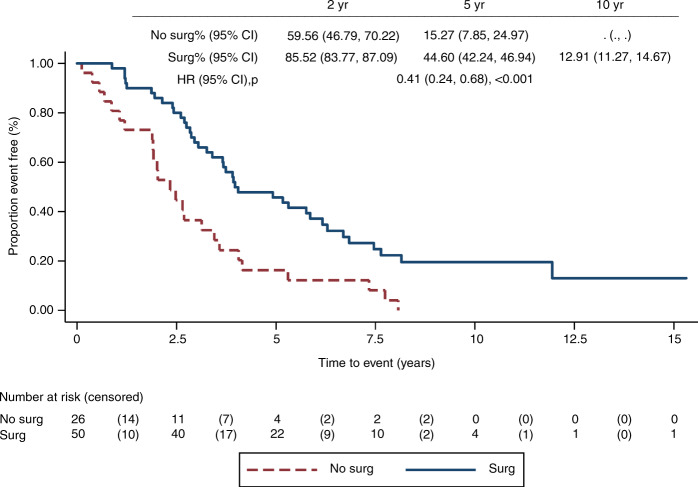
Kaplan-Meier plot of overall survival by surgical category. OS by surgical type (dnMBC patients only); reference category: no surgery.

Amongst dnMBC patients, 65.8% (50/76) had local surgery. Survival was better in those who had surgery, with a univariable HR of 0.41 (0.24–0.68, *p* = <0.001) and 5-year OS of 44.6% (42.24–46.94) vs. 15.27% (7.85–24.97) (Fig. 3). Patients were treated with palliative cytotoxic chemotherapy in 98.7% of the dnMBC group vs. 71.4% in the early-12 group, and 70.3% in the late-relapse group. Palliative hormone therapy was also the highest in the dnMBC group (68.4%), whereas it was given in 28.6% of patients who relapsed within 12 months and 61.4% of patients with a late relapse. Palliative radiotherapy was also administered at the highest rate in the dnMBC group, with 76.3% of patients receiving it, compared with 60.0% in the early-12 group and 50.0% in the late-relapse group.

## Discussion

This is the largest prospective study to evaluate metastatic disease in the young-onset breast cancer population. We have shown that young women who develop secondary metastatic disease, even if greater than 5 years after diagnosis, have shorter survival time following diagnosis of metastases, compared with those who present with de novo metastatic disease. When survival from initial diagnosis (OS) was compared, this was superior for those with dnMBC, compared with those who developed relapsed disease within 24 months.

In this study, nearly a third (27.1%) of women developed metastatic recurrence after presenting with localised disease. Only 2.6% of this cohort had metastatic disease at presentation, lower than the national (unselected age) estimate of 6–7% from Cancer Research UK.^1^ Late stage at diagnosis is reported to be more common in women aged greater than 80, and so this likely contributes to the higher figure nationally.^1,13^ It is also possible that there was an element of selection bias, as oncologists may have chosen to recruit patients with metastatic disease to an interventional study rather than an observational one (although participation in an interventional study did not exclude patients from this study). However, a retrospective Swedish study found that only 1% of patients aged less than 40 presented with metastases, with this figure increasing successively in each age cohort (up to 10% for those aged greater than 80).^13^ In other retrospective studies of women in the same age group, the de novo rate was 3.0–3.9%, not dissimilar to what is reported here.^15–17^

With regard to the identification of de novo disease, at the time of recruitment, further preoperative imaging would only have been performed if the patient had symptoms suggestive of metastatic disease, or possibly because of clinically positive axillary nodes or a large primary tumour. The 2009 National Institute of Clinical Excellence guidelines advised that patients with early breast cancer should only undergo staging for metastatic disease in the presence of symptoms.^18^ CT would not have routinely been used in all centres; screening for occult metastases may instead have involved chest radiographs, liver ultrasound and bone scintigraphy.^19^ Therefore, women diagnosed with de novo disease at the time of the POSH study are more likely to have had adverse tumour features clinically or concerning symptoms. Given that we have shown younger patients to have a high rate of node positivity and more advanced T stage, in addition to ER negativity, it is possible that they were more likely to have baseline imaging. At the present time, there is no difference in recommended staging or follow-up for younger patients.^11^ One retrospective study found that a baseline PET/CT scan upgraded 15% of young, asymptomatic patients with early-stage breast cancer to stage IV.^20^ Given the better survival for de novo patients here, compared with those who relapsed within 24 months, and the more adverse biology in young patients, age should be incorporated into clinicians’ decision-making with regard to baseline imaging. A randomised controlled trial would be required to identify whether routine imaging for metastatic disease at baseline would improve survival for young patients.

A third of women who developed metastatic disease (34.1%) relapsed within 24 months of diagnosis. This group had a significantly worse OS compared with those who presented with metastatic disease. A review of long-term survivorship with metastatic disease shows 10-year OS rates of 12.45% (95% CI 5.81–21.76) for dnMBC patients, but only 1.73% (0.55–4.24) for patients who relapsed within 24 months (Fig. 1b). For PDRS, outcomes were better for de novo disease compared with all relapsed groups, including those who relapsed late (after 5 years). Whether the improved outcomes for dnMBC are due to inherently more favourable biology or different treatment approaches is unclear. Adjuvant therapy is likely to drive subclonal diversification, resulting in mutations that confer resistance to further cytotoxic/hormonal treatment. Women with de novo disease may also have been treated more aggressively, e.g., with local therapy for oligometastatic disease. It might be assumed that late-relapsing disease (84.2% of whom are ER-positive) is inherently indolent, but this study challenges that assumption. Either the metastatic clones present remain dormant until acquiring sufficient oncogenic drivers, or the clones are present from diagnosis, but are suppressed by adjuvant hormone therapy. Further genomic work to understand the subclonal architecture, and heterogeneity of primary and metastatic sites, is required, in order to guide treatment strategies in the era of personalised medicine.

The de novo cohort had a remarkably high prevalence of HER2-positive tumours (47.9%). The number of HER2 + cases in this group may contribute to the improved survival seen with dnMBC, given previous reports of long-term responses to trastuzumab in a proportion of patients with HER2 + MBC.^21^ However, not all HER2 + patients received trastuzumab, reflective of the era during which POSH was recruited. Trastuzumab was approved for use in the adjuvant setting in 2005, 3 years after the trial began recruitment. In general, baseline tumour characteristics were adverse in the early-relapse group, with the greatest proportion of T2/3, node-positive and LVI tumours. These features may account for the worse prognosis in this group. The late-relapse group was marked by ER positivity (84.2% vs. 41.4% in the early-12 group), node negativity (31.4% vs. 16.2%) and smaller median tumour size (25 mm vs. 32 mm).

It has been hypothesised that there is a different pattern of metastatic spread between patients with primary and secondary metastatic breast cancer. In fact, the de novo group had the highest proportion of widespread (bone, visceral and brain) metastases during their disease course (26.3%), although this may be reflective of their longer survival and the resultant time to allow dissemination. In addition, their widespread metastases may have produced symptoms that resulted in their de novo disease being detected with imaging at diagnosis. The prevalence of HER2 positivity in this group may also account for this. The late-relapse group was the least likely to develop brain metastases during the course of their disease (24.3%); proportions were similar for the dnMBC and early-12 groups (39.5 and 38.6%, respectively). However, only one of the dnMBC patients (1.3%) had brain metastases at diagnosis, compared with 18.6% of the early-12 group. This may reflect clinicians being more likely to perform a baseline CT brain in patients with recurrent, as opposed to newly diagnosed, breast cancer. Young age has previously been associated with an increased risk of brain metastases; the prevalence in this entire cohort was nearly a third (32.3%).^11^ Clinicians should be vigilant for central nervous system symptoms in young women during follow-up for breast cancer. There is no clear evidence that the sites of distant disease explain the differing prognosis between relapse categories; it seems more likely that the underlying biology influences metastatic sites, which determines whether or not a patient presents with de novo disease.

This cohort is unique not only for its age but also for completeness of *BRCA* gene mutation testing. It was notable therefore that a relatively large proportion of patients with de novo disease (11.8%) had a *BRCA2* mutation, whereas just one (1.3%) had a *BRCA1* mutation. Although the 69.3% ER-positivity rate in this group may explain this to some extent, the ER positivity was higher in the early-24–60 and late-relapse groups with a lower BRCA2 prevalence (5.8% and 6.3%, respectively). Across the cohort of 862 patients with metastatic disease, the BRCA2 mutation rate was 5.6%; the rate was 5.0% across the POSH cohort as a whole (excluding dnMBC patients).^22^ The reason for such a large proportion of dnMBC cases having a *BRCA2* mutation is unclear; it is possible that a family history of breast and ovarian cancer in *BRCA2* mutation carriers meant that they were more vigilant regarding symptoms of metastatic disease. In addition, 57% of the dnMBC cohort had bone metastases at presentation (in common with 69.3% being ER + ve, the common phenotype arising from a *BRCA2* mutation); perhaps, bone pain in a young woman is a red- flag symptom that resulted in early imaging. This might enrich the dnMBC with *BRCA2* mutation carriers. Our results would suggest that further studies using *BRCA* germline testing in young women with dnMBC are warranted.

Primary surgery in patients with de novo metastatic breast cancer remains a debated issue, with decisions made on a case-by-case basis. In this study, for patients with dnMBC, there was an improved survival for those who had surgery (*n* = 50) vs. those who did not (*n* = 26), with a univariable HR of 0.41 (*p* = <0.001). This outcome is susceptible to selection bias as locoregional treatment was presumably more likely to occur if disease was apparently relatively indolent, less widespread and the patient’s performance status was good. A randomised prospective trial to address this issue in the modern era is required.

The groups least likely to receive palliative chemotherapy were the early-12 and late-relapse groups (71.4% and 70.3%, respectively). In the early-12 group, this may be related to lack of recovery from toxicity following recent adjuvant/neoadjuvant chemotherapy, radiotherapy and surgery. Physicians may also have chosen not to treat refractory disease with further systemic treatment. Amongst the late-relapse group, 84.2% of whom were ER-positive, most patients received hormone therapy (61.4%). This is likely to reflect the burden and distribution of metastatic disease. It may also be that the late relapses were perceived as more indolent. They experienced less brain metastases compared with the dnMBC group (24.3% vs. 39.5%), but a similar proportion of visceral metastases (77.9% vs. 75.0%). In fact, the poor PDRS of the late-relapse group compared with dnMBC would indicate that alternative treatment strategies are needed for this cohort.

The potential limitations of this study include its age. As a result, there have been changes in systemic options available, although most patients in this study were treated with anthracycline + /− taxane chemotherapy, and approximately half of them received trastuzumab if HER2 + .^12^ There is an increasingly proactive approach to staging investigations, including the use of advanced imaging, such as positron emission tomography. This may mean that more patients are now diagnosed with occult metastases at presentation, affecting the characteristics of the de novo group. Finally, we cannot rule out a degree of selection bias during POSH recruitment. However, as detailed previously, POSH participants recruited from England represented 23% of the available population during the recruitment period, and comparison with cancer registry data confirmed that the cohort is representative of the wider population.^22^ The strengths of this study include the large cohort size and complete germline *BRCA* testing. There are few missing data (with HER2 status missing in only 5.7% of cases) and long follow-up, with only a small number of patients lost to follow-up.

## Conclusion

This is the first report to describe patterns of metastatic disease in a large prospective cohort of young-onset breast cancer patients, with a long follow-up period and complete *BRCA* germline testing. We have shown that women aged 40 or less with de novo metastatic breast cancer have better survival following the onset of metastatic disease than those who develop secondary breast cancer. Despite more favourable baseline tumour characteristics, patients who developed late-onset metastatic disease had a worse PDRS than de novo patients, indicating that chemotherapy-resistant clones and/or perceived poor fitness due to prior therapies have a significant impact on prognosis. A notable proportion of women with dnMBC had a germline *BRCA2* mutation; this has not previously been highlighted in the literature.

## Supplementary information

Supplementary Information

## Data Availability

The data sets generated and/or analysed during this study are not publicly available.
