# Supplementary material for: Survival and disease characteristics of de novo versus recurrent metastatic breast cancer in a cohort of young patients
Source: Br J Cancer. 2020 Mar 31;122(11):1618–29. doi: 10.1038/s41416-020-0784-z (PMC7250836; doi:10.1038/s41416-020-0784-z)
Supplement: Supplementary file 1 — Supplementary Information [file 41416_2020_784_MOESM1_ESM.docx]

Supplementary Figures


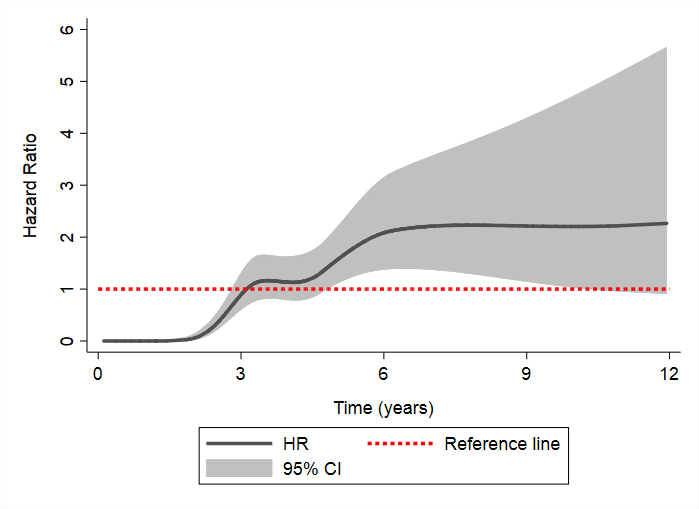


Supplementary Figure 1A. Time-varying HR for OS for dnMBC vs. early24 to 60; reference category: dnMBC.


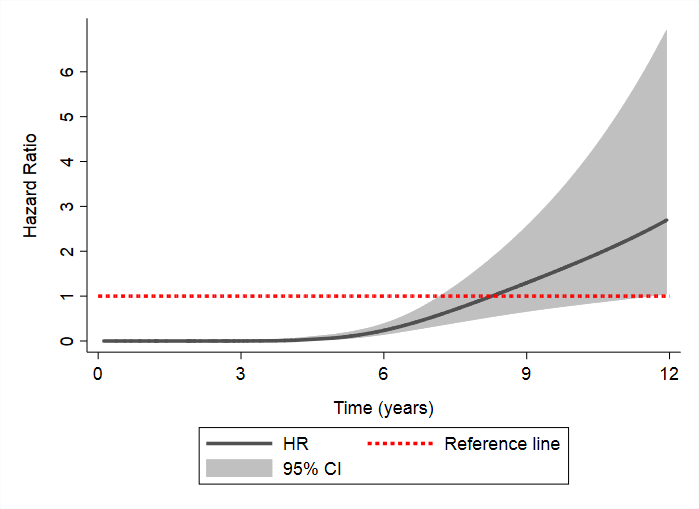


Supplementary Figure 1B. Time-varying HR for OS for dnMBC vs. late60+; reference category: dnMBC.

**Supplementary Information**

**Statistical Analysis Plan for the POSH study (Prospective Study of Outcomes in Sporadic versus Hereditary breast cancer) – Mo vs M1 paper**

*[Please note this statistical analysis plan has been written in the past tense because it will form the basis of a paper]*

The headings used in this document come from the STROBE reporting guideline for observational studies (see <http://www.strobe-statement.org/>.

**Statistical Analysis Plan Version**

| **Issue no** | **Revision History** | **Author** | **Date** |
| --- | --- | --- | --- |
| 0.1 | First draft | Ellen Copson and Hayley McKenzie | 12^th^ April 2018 |
| 0.2 | Updated following meeting with Ellen Copson, Hayley McKenzie, Tom Maishman and Laura Day | Tom Maishman and Laura Day | 12^th^ July 2018 |

**To be approved and reviewed by:**

|  | **Name** | **Date** |
| --- | --- | --- |
| **To be reviewed by:** | Diana Eccles, Peter Simmonds and Ellen Copson |  |
| **To be approved by:** | Diana Eccles |  |

1. **Introduction**
   1. **Background / Rationale**

Breast cancer is the most common neoplasm in women, with a yearly incidence of over 55,000 in the UK(1). When localized to the breast and lymph nodes it is often curable, but for the 3 to 6% who present with *de novo* metastatic breast cancer (dnMBC) it is not likely (2, 3). Overall, the median survival of those with metastatic breast cancer (MBC) is 2 to 3 years(4), although the range is wide, with some patients with ER+ or HER2+ disease living much longer.

Most studies to date have shown a longer survival time for those presenting with DNMBC compared to those who develop recurrent MBC (5-8). In one study this longer survival time was limited to patients with a metastasis-free interval of greater than 24 months; those with a shorter interval had a significantly worse prognosis(9). The phenotype of breast cancer for those with DNMBC is unclear. Some studies have reported more favourable pathological features(5), while others have reported an aggressive phenotype in those with *de novo* MBC(3). Data regarding clinical presentation has also yielded conflicting results; some reports suggest a similar pattern of metastatic disease(5), while others show a lower prevalence of brain metastases compared to those with recurrent disease(7, 9). Locoregional management in patients with DNMBC is an ongoing debate; results from randomised trials have been conflicting(10). Therefore, the clinicopathological presentation, prognosis and optimal treatment for patients with *de novo* metastatic breast cancer are not yet fully elucidated.

Women aged less than 40 at diagnosis are more likely to have breast cancer with adverse biological features, including higher grade, absence of hormone receptors, lymph node involvement and vascular invasion(11). We plan to characterise the clinical features, pattern of disease progression and survival of young breast cancer patients who present with metastatic disease, compared to those who later develop distant metastases in a large prospective cohort of young patients genotyped for germline BRCA1/ 2 and other high and moderate penetrance breast cancer susceptibility genes.

- 1. **Objectives**

This paper presents the results from analyses carried out on data collected from the POSH study. To ensure clarity is provided in the objectives, the following definitions are provided below:

**Definitions:**

- dnMBC (de Novo Metastatic Breast Cancer)=M1 stage patients
- early24=M0 stage patients who have experienced a distant relapse within 24 months
- early12=M0 stage patients who have experienced a distant relapse within 12
- months
- early24_to_60=M0 stage patients who have experienced a distant relapse after 24 months but within 60 months
- early60+=M0 stage patients who have experienced a distant relapse after 60 months

The primary objective was to:

- To compare Overall Survival (OS), defined as time from date of diagnosis to death from any cause, of early12 with that of dnMBC patients.

Secondary objectives:

- To compare OS of early24 with that of dnMBC patients.
- To compare Post Distant Relapse Free Survival (PDRS), defined as time from date of distant metastases to death from any cause, early12 with that of dnMBC patients.
- To compare PDRS, defined as time from date of distant metastases to death from any cause, early24 with that of dnMBC patients.
- To describe patient and tumour characteristics (including Body Mass Index [BMI], BRCA status and sites of metastatic disease) and clinicopathological features of primary tumours in M distant groups:
  - dnMBC
  - early12
  - early24
  - early24_to_60
  - early60+
  - All patients

(with formal comparisons between dnMBC v early12, and also between dnMBC v early24).

- dnMBC patients only: To compare OS in dnMBC patients who had breast conserving surgery (BCS) or mastectomy (or nodal surgery) vs those who had no surgery.
- dnMBC patients only: To compare PDRS in dnMBC patients who had BCS or mastectomy (or nodal surgery) vs those who had no surgery.
- M0 stage patients only i.e. non-dnMBC: To assess the correlation between MFI (metastasis-free interval), defined as time between date of diagnosis and date of first distant relapse, and PDRS in M0 patients who later develop metastatic disease.

1. **Methods**
   1. **Study Design**

The POSH study is a prospective cohort study. The protocol for the study can be found in the following journal article <http://www.biomedcentral.com/1471-2407/7/160>.

- 1. **Setting**

The POSH study recruited women from breast centres across England, Scotland, Wales and Northern Island between 1^st^ June 2001 to 31^st^ January 2008.

- 1. **Participants**

The study recruited ≈3000 women aged 40 years or younger at breast cancer diagnosis. The women had to have been diagnosed with breast cancer between January 2000 and January 2008. In addition, 43 women aged 41-50 were included if they had a known BRCA1 or BRCA2 gene mutation and were diagnosed with invasive breast cancer within the study period but were excluded for this analysis. Women were excluded if they had a previous invasive malignancy (with the exception of non-melanomatous skin cancer), were not available for follow up or refused consent to retain diagnostic and follow up data. A total of 2977 women were included in the analysis population.

Clinical follow up data were obtained from the patient medical records by the clinical trials practitioner (CTP) at each recruiting centre. Data forms collecting information at diagnosis, 6 months, 12 months were completed by the CTP usually at 12 months from diagnosis. Annual data collection was continued from the date of definitive diagnosis until death, loss to follow up or until the end of the current phase of the study (June 2016).

- 1. **Variables**

| **Variable** | **Type of data / categories** | **Amount of missing data** | **Possible reasons for missing data** |
| --- | --- | --- | --- |
| **Primary outcome** | | | |
| Time to death (OS) , in years | Survival data  Date of death from any cause – Date of invasive breast cancer diagnosis | N/A, patients who haven’t experienced an event will be censored at the date of their last follow up visit | N/A |
| **Secondary outcomes** | | | |
| Time from first relapse to death from any cause (PDRS) | Survival data  Date of death (or last follow-up if not died) – Date of distant relapse | N/A, patients who haven’t experienced an event will be censored at the date of last follow-up. | N/A |
| **Candidate predictor(s)** | | | |
| M stage | Multiple binary categories:   - deMBC vs. early12 - deMBC vs. early24   Also categorical:   - deMBC - early12 - early24 - early24_to_60 - early60+ | 26 records | Consider Missing Completely at Random (MCAR) |
| Surgical Indicator | Binary (BCS or Mastectomy or Nodal surgery only vs. No surgery) | 0 records | N/A |
| **Potential confounders / effect modifiers** | | | |
| 1. Age at diagnosis, in years | Continuous, in years  In addition, Categorical:  18 to 25, 26 to 30, 31 to 35, 36 to 40 | 0 records | N/A |
| 1. Duration of follow-up, in years | Survival data (reverse Kaplan-Meier of OS)  Date of last follow-up – date of diagnosis.  Patients who die from any cause are censored at the date of last follow-up. | 0 records | N/A |
| 1. Ethnicity | Categroical (Caucasian/White, Black, Asian, Other, or Missing/unknown) | 40 records | Consider MCAR |
| 1. Body mass index | Categorical (Underweight/Normal, Overweight, Obese) | 116 records | Patients who had not had chemotherapy were less likely to have their weight or height measured. In addition, some patients might have had chemotherapy privately and not had their weight or height recorded. Assume MAR. |
| 1. Family History (patients with at least one first or second degree relative with breast cancer) | Categorical  Yes, no, or missing/unknown. | 122 records | MCAR |
| 1. Presentation | Categorical  Symptomatic, screen-detected, other, or missing/unknown | 14 records | MCAR |
| 1. Histological grade (Grade) | Categorical  1, 2, 3, or not graded/missing/unknown | 89 records | Consider MAR |
| 1. Histological type | Categorical  Ductal, lobular, ductal & lobular, mixed, medullary, metaplastic, other, unclassified, not graded, or missing/unknown | 50 records | Consider MAR |
| 1. Focality of cancer | Categorical  Multifocal, localised, or missing/unknown | 297 records | Consider MAR |
| 1. ER status | Categorical  Negative, positive, or missing/unknown | 18 records | MCAR |
| 1. HER2 status | Categorical  Negative, positive, or missing/unknown | 350 records | Missing when diagnosis predated routine testing. Potential bias towards missing in patients not experiencing disease recurrence. Consider MAR for this analysis. |
| 1. PR status | Categorical  Negative, positive, or missing/unknown | 584 records | Consider MAR |
| 1. Pathological N stage (lymph node status) | Categorical  N0, N1 or missing/unknown | 29 records | No axillary surgery, no lymph nodes in resected specimen. Consider MAR |
| 1. Lymphovascular Invasion | Categorical  Absent, present, or missing/unknown | 229 records | Consider MAR |
| 1. Number of positive axillary lymph nodes | Continuous (integer) | 76 records | Consider MAR |
| 1. Maximum tumour diameter invasive, in cm | Continuous | 199 records | Consider MAR |
| 1. Maximum tumour diameter overall (including ductal carcinoma in-situ) (pathological), in mm | Continuous | 157 records | Consider MAR |
| 1. Maximum tumour diameter in-situ, in mm | Continuous | 2588 records | Consider MAR |
| 1. Pathological T stage (for patients receiving neo-adjuvant chemotherapy) | Categorical  T0, T1, T2, T3, T4, Tis, Tx, or missing/unknown | 119 records | MCAR |
| 1. Chemotherapy timing | Categorical  Adjuvant, neo-adjuvant, palliative, or not applicable | 0 records | N/A |
| 1. Adjuvant trastuzumab | Categorical  Yes, no/missing/unknown | 2609 records | MAR |
| 1. Adjuvant Radiotherapy | Categorical  Yes, no/missing/unknown | 2380 records | MAR |
| 1. Hormone treatment | Categorical  Yes, no/missing/unknown | 1132 records | MAR |
| 1. Year of diagnosis | Categorical  2000, 2001… , 2008 | 0 records | N/A |
| 1. Patient status (for PDRS) | Categorical:  Alive following distant relapse, censored at last follow-up  Dead following distant relapse | 0 records | N/A |
| 1. Censored variable (for PDRS) | Binary (0,1)  0=Did not experienced death after distant relapse  1=Experienced death after distant relapse | 0 records | N/A |
| 1. Patient status (for OS) | Categorical:  Alive, censored at last follow-up  Dead | 0 records | N/A |
| 1. Censored variable (for OS) | Binary (0,1)  0=Did not experienced OS event  1=Experienced OS event | 0 records | N/A |

- 1. **Data sources/measurement**

The tumour biopsy, definitive histopathological report, clinical and radiological reports were all submitted to the study. Pathological characteristics of the tumours were taken from the diagnostic and surgical histopathology report, or clinic letters if not available, clinical staging from the clinical and radiological reports or clinical letters where above not available. For patients treated with neoadjuvant chemotherapy radiological tumour size was used as in previous papers.

National death data were obtained for patients in the cohort from the Medical Research Information Service (MRIS).

ER, PR and HER2 data are taken from pathology reports. Scoring systems varied as expected across contributing hospitals. Positive and Negative categories are straightforward however borderline results exist in all three IHC categories and were classified as positive or negative. The borderline category was merged with positive for the purposes of these analyses.

HER2 data: There are concerns regarding the amount of missing HER2 data obtained. This is covered in more detail in the BRCA v1 SAP. Her2 was not routinely accessed or recorded at the start of the study. However if patients diagnosed prior to this date relapsed, then where the primary tumour was reassessed the information was added to the primary tumour data.

This paper presents the results of analyses conducted on follow up data available up until 26 June 2016.

- 1. **Representativeness**

Clinical data for all patients were collected via standard clinical research forms which were completed from the clinical notes by the Clinical Trials Practitioner in each centre, and by copies of original pathology, scan reports and medical notes*.*

Recruiting bias: Possibly patients presenting with metastases were not invited (no evidence of this). Possibly patients who just had surgery for very small tumours were not recruited since we recruited mainly through oncology clinics.

Survival bias: Patients who were diagnosed with invasive breast carcinoma but died before consenting to the trial were not included in the trial. As a result, this could lead to a healthier sample of the UK population being analysed for the POSH cohort.

- 1. **Study Size**

This is covered in the BMC paper.

- 1. **Statistical Methods**

**Patients excluded from the analyses**

Patients were excluded from this analysis if they were 41 years of age or over at the date of invasive breast cancer diagnosis (43 patients) i.e. a patient born on 01-Jan-1960 would be included if she was diagnosed before 01-Jan-2001 and excluded if she was diagnosed on or after 01-Jan-2001. Patients were also excluded if they were diagnosed outside of the study period (n=1), did not have invasive cancer (n=72) or did not have any follow-up/primary tumour information available (n=2). As a result, a total of 2977 patients were included in the analysis population.

**Primary Endpoint**

1. Overall Survival (OS), where OS is defined as time from date of diagnosis of primary breast cancer to date of death from any cause.

**Secondary Endpoints**

The secondary endpoints are:

1. Post Distant Relapse Free Survival (PDRS) defined as time from date of diagnosis of distant metastatic disease to date of death from any cause.

**Statistical analyses**

1. We described the baseline patient and tumour characteristics of the cohort by M categorisation:

Patient characteristics:

- Age at diagnosis, in years – median (range, IQR), n(%);
- Age at diagnosis (18-25, 26-30, 31-35, 36-40) – n(%);
- Ethnicity (Caucasian/White, Black, Asian, Other, or missing/unknown) – n(%);
- Obesity (BMI) – median (range, IQR), n(%);
- Family History (yes, no, or missing/unknown);
- Presentation (Symptomatic, screen-detected, other, or missing/unknown) – n (%);
- Genotype (BRCA1+, BRCA2+. TP53+ , other cancer susceptibility gene (CSG) mutation, sporadic)

Tumour characteristics:

- Histological grade (1, 2, 3, not graded/missing/unknown) – n (%);
- Histological type (Ductal, lobular, ductal & lobular, mixed, medullary, metaplastic, other, unclassified, not graded, or missing/unknown) – n (%);
- Surgical margins (0, >0 and <1, ≥1 and ≤5, >5, or missing/unknown) – n (%);
- Extensive in situ (EIC) component (EIC Positive, EIC Negative, or missing/unknown) – n (%);
- Lymphovascular invasion (Absent, Present, or missing/unknown) – n(%);
- Number of positive axillary lymph nodes (0, 1-3, 4-9, 10+, or missing/unknown) – n(%);
- Oestrogen receptor (ER) status (negative, positive, or missing/unknown) – n (%);
- Progesterone receptor (PR) status (negative, positive, or missing/unknown) – n (%);
- Human Epidermal growth factor receptor 2 (HER2) status (negative, positive or missing/unknown) – n (%).
- Focality of cancer (multifocal, localised, or missing/unknown) – n (%);
- Pathological T stage (T0, T1, T2, T3, T4, Tis, Tx, or missing/unknown) – n(%);
- Pathological N stage (N0, N1, or missing/unknown) – n (%);
- Maximum diameter invasive tumour, in mm – median (range, IQR), n(%), missing/unknown – n(%).

1. We described the primary oncological treatment of the cohort by treatment type
   - Breast surgery median (definitive type: BCS/ mastectomy– n(%);
   - Axillary clearance/sample details (only if available at the time of analysis) (yes or no/missing/unknown) – n(%).
   - Palliative Chemotherapy – n(%);
   - Palliative trastuzumab (yes or no/missing/unknown) – n(%);
   - Palliative Radiotherapy (yes or no/missing/unknown) – n(%);
   - Hormone treatment (yes or no/missing/unknown) – n(%);
2. We produced Kaplan-Meier survival curves for OS and PDRS for the following groups and provided Hazard Ratios (except for the all patients group):
   - All patients;
   - dnMBCvs. early12
   - dnMBCvs. early24
   - dnMBCvs. early24_to_60
   - dnMBCvs. early60+
3. For deMBC patients only: We produced Kaplan-Meier survival curves for OS and PDRS for the following groups and provided Hazard Ratios:
   - Surery vs. no surgery.
4. For M0 stage patients only: We assessed the correlation between MFI and PDRS.
5. We fitted a multivariable model for OS and PDRS for the following covariates to compare dnMBCvs. early12 and dnMBCvs. early24, with the following covariates fitted:

- Age at diagnosis, in years (fitted as a continuous covariate);
- Body Mass Index (fitted as a categorical covariate [Underweight/Healthy, Overweight or Obese]);
- Histological Grade (fitted as a categorical covariate [1, 2 or 3]);
- Maximum invasive tumour size, in mm (fitted as a continuous covariate);
- N stage (fitted as a binary covariate [N0 or N1]);
- ER status (fitted as a binary covariate [Negative or Positive]);
- HER2 status (fitted as a binary covariate [Negative or Positive]);
- Ethnicity (fitted as a categorical covariate [Caucasian, Black or Asian]);
- Surgery vs. no surgery.

Hazard Ratios

Evidence suggests that the effect of ER status on the HR for relapse changes over time (Azzato, et al, 2009, Bellera et al, 2010)^1^. Indeed, this was evident after testing the proportional hazards assumption based on the Schoenfeld residuals and using the identity matrix for the time-scaling function^2^. This result provided strong evidence against the Cox proportional hazards assumption, which was also seen when plotting the scaled Schoenfeld residuals over time^2^. We therefore stratified any Cox models by ER status.

**Method used to handle missing data**

This was a complete case analysis.

**Appendix 2**

**Example of Consort Flow diagram**

Eligible but excluded from this analysis (n=44):
Gene carriers aged 41-50 (n=42)
Missing primary tumour data (n=2)

Excluded as ineligible (n=74):

- Diagnosed outside of the study period (n=1)
- No invasive breast cancer (n=72)
- Non-mutation carrier aged 41-50 (n=1)

Patients aged 40 years or younger at diagnosis included in analysis

n=2977

Total number of patients recruited to the POSH study

n=3095

Satisfying eligibility criteria

n=3021
